# A Sad Case

**Published:** 1881-03

**Authors:** 


### A SAD CASE.

Nomenclature of Diseases of the Royal College of Physicians.—A committee of thirty, including some of the most prominent medical men of London and vicinity, has been appointed to superintend the decennial revision of the nomenclature of diseases.—*Medical Record.*

In 1869 these learned men, sitting in solemn conclave, concluded that the non surgical "ills which human flesh is heir to," were just 901! Now they propse to increase our woes and add a few *names* to the list.

Theirs is a sad, sad case; but sad as it is there is a remedy—pure homœopathy. Try it, gentlemen.

